# Arm-pull thrust in human swimming and the effect of post-activation potentiation

**DOI:** 10.1038/s41598-020-65494-z

**Published:** 2020-05-21

**Authors:** Tiago M. Barbosa, Jia Wen Yam, Danny Lum, Govindasamy Balasekaran, Daniel A. Marinho

**Affiliations:** 1 Physical Education and Sport Science Academic Group, National Institute of Education, Nanyang Technological University, Singapore, Singapore; 2 Department of Sport Sciences, Polytechnic Institute of Bragança, Bragança, Portugal; 3 Research Centre in Sports, Health and Human Development - CIDESD, Vila Real, Portugal; 4 Sport Science and Sport Medicine, Singapore Sport Institute, Singapore, Singapore; 5 Department of Sport Sciences, University of Beira Interior, Covilhã, Portugal

**Keywords:** Behavioural methods, Behavioural methods, Biomedical engineering, Biomedical engineering

## Abstract

The aim of this study was to analyse the front-crawl arm-pull kinetics and kinematics, comparing it before and after post-activation potentiation (PAP), and the associations between variables describing of the arm-pull kinetics. Twelve male competitive swimmers were randomly assigned to perform two different warm-ups in a crossover manner: (i) non-PAP (control condition); and (ii) PAP (experimental condition). PAP consisted of 2 × 5 arm-pulls with resistance bands by both upper-limbs. Eight minutes later, participants underwent a 25 m all-out trial in front-crawl arm-pull. Kinetics (i.e., peak thrust, mean thrust and thrust-time integral) and kinematics (i.e., speed and speed fluctuation) were collected by an in-house customised system composed of differential pressure sensors, speedo-meter and underwater camera. There was a significant and large improvement of the arm-pull kinetics after completing the warm-up with PAP sets (0.010 < *P* < 0.054, 0.50 < d < 0.74). There were non-significant and small effects of PAP on speed (*P* = 0.307, d = 0.18) and speed fluctuation (*P* = 0.498, d = 0.04). Correlation coefficients among kinetic variables were significant with large associations (0.51 < R < 0.90, 0.001 < *P* < 0.088). In conclusion, warm-ups including PAP conditioning sets elicit a large improvement in the thrust, but with small improvement in performance. Variables used to characterise thrust are strongly correlated and hence can be used interchangeably.

## Introduction

Swimming is one of the most challenging locomotion techniques for humans. Human swimming is a multifactorial phenomenon, encompassing several determinants. Acceleration of human swimming is the net balance between drag force and thrust, taking into account the inertial term:

1 $$a=\frac{T-D}{m}$$

where *a* is the body’s acceleration, *T* the thrust, *D* the drag force and *m* the total mass. Equation 1 can be decomposed as:

2 $$a=\frac{({T}_{h}+{T}_{f})-({D}_{f}+{D}_{p}+{D}_{w)}}{(BM+{m}_{a})}$$

where *a* is the acceleration, *T*_*h*_ the thrust by the hands or upper-limbs, *T*_*h*_ the thrust by the feet or lower-limbs, *D*_*f*_ the friction drag, *D*_*p*_ the pressure drag, *D*_*w*_ the wave drag, *BM* the body mass and *m*_*a*_ the added mass of water. Researchers have been conducting studies to understand the effect of drag force in human swimming by numerical simulations^1^, experimental testing^2^ and analytical procedures^3^. An interesting debate on how to measure human thrust is ongoing^4,5^. In comparison, the amount of research on the thrust production is far more limited. Nevertheless, researches making use of numerical simulations^6,7^, experimental testing^8^ and analytical procedures^9^ are available in the literature.

Most experimental testing protocols were conducted with robotic arms or legs mimicking the arm-pull or kicking, respectively^8,10^. These studies came in the wake of researches on propulsion in aquatic animals that also used bio-robots mimicking the motion, for instance, by fish^11^ or frogs^12^. Even though these studies provided valuable insights on the propulsive mechanisms in human swimming, the ecological validity of these findings are somehow limited. One can argue that despite their best attempt to mimic as much as possible the motion of human limbs swimming, this is not completely possible to be done based on these days’ technological advances. To tackle this concern, alternatively, it is possible to use tethered swimming^13^. In this evaluation technique, a human swimmer is invited to undergo an in-water testing session where he will swim fully or partially tethered by a string at waist level. The other end of the string is attached to a strain gauge, load cell, or any other sensor that can measure changes in the string tension. Although this technique assesses a human swimmer, some concerns have been raised on the water flow surrounding the body when tethered. When tethered, the stationary swim may affect the water flow around the swimmer’s body, and therefore interfering with propelling efficiency. Arguably, the transfer of kinetic energy to water is larger when tethered, leading to less ability to produce mechanical power to overcome the drag force. Altogether, these two techniques have advantages that cancel out the limitations of each other.

Ideally, there should exist a technique that would enable the assessment of humans in unrestricted swimming. The best option fulfilling these criteria is placing sensors on the propelling limbs of humans. A few studies have reported the use of differential pressure sensors^14–16^. Some of the procedures using differential pressure sensors are time-consuming, encompassing a heavy set-up, constraining the swimmer’s technique and a taxing data handling^14^. These challenges led to studies recruiting one to six swimmers^14,17^. Meanwhile, a straightforward and user-friendlier procedure is also available^18^. The use of this system or its components can facilitate the recruitment of a larger number of swimmers, providing a larger dataset with a better external validity. As far as our understanding goes, such report of human arm-pull thrust using differential pressure sensors during unrestricted swim in a large sample of swimmers cannot be found.

A concurrent interest is how to enhance the thrust production. Recent reports noted that the phenomenon of post-activation potentiation (PAP) can trigger a performance enhancement. PAP conditioning sets can be included as part of the warm-up routine before a race. After undergoing a PAP conditioning set, there is an increase in the muscle isometric twitch and low frequency tetanic^19^. I.e., the force exerted is increased due to its previous contraction during the PAP set. In the PAP set, the subject is requested to perform a high intensity resistance exercise via dynamic or isometric contraction. After sometime, known as latency period, the main exercise bout is performed^20^. Because there is a lag period between the main exercise bout (e.g. a sprint) and the conditioning set, PAP is most of the times incorporated in the warm-up routine. In human swimming, PAP may help to enhance the sprinting performance^21–23^; with mostly small effects sizes^24^. One study reported that PAP can induce an increase in the flutter kick thrust^16^. However, human kicking contributes to just 10–15% of overall swim speed^25–27^. Remaining 85–90% of the speed is due to arm-pull thrust. Thus, it remains to be investigated if a PAP conditioning set can enhance the arm-pull thrust and the swimming performance. If so, it might be possible to set a mechanistic relationship between PAP, thrust, swim kinematics and performance.

Assessing or monitoring the thrust in competitive swimming, it is a standard procedure to do the decomposition of the thrust-time curve. Likewise, this decomposition is a common practice in other settings where time-series of kinetic variables are collected, such as force-time or pressure-time. For instance, as the decomposition of the ground reaction force^28^ or the plantar pressure of the human gait^29–31^. In one of these studies, it was noted that the variables commonly used to characterise plantar pressures were highly inter-correlated (0.78 < R < 0.90)^29^. Hence, a smaller set of parameters may be more efficient for capturing the biomechanical behaviour of interest. I.e., plantar pressure parameters (peak, mean and impulse) can be reasonably compared across parameters, the authors argue. A systematic review concluded that reporting the impulse added limited value to the findings, as in the case when studying diabetic foot^31^. In the case of human swimming, there is not a conventional or standard procedure on the variables to be selected. Researchers reported on regular basis either the peak thrust, mean thrust or thrust-time integral. It is yet unclear to which extend these variables can be used, reported and interpreted interchangeably. If a strong association does exist, as in human gait on-land, the report of a smaller set of variables can be of interest for some researchers and practitioners. Thus, such findings can clarify this issue and help setting up a standard procedure on the variables to be reported when assessing human thrust.

The aim of this study was to: (1) analyse the front-crawl arm-pull kinetics and kinematics, comparing it before and after PAP, and (2) the associations between variables describing of the arm-pull kinetics. It was hypothesised that: (1) following PAP conditioning sets, the arm-pull kinetics as well as kinematics would improve and, as such, the performance; and (2) there would be a strong association among different kinetic variables.

## Results

**Figure 1:**
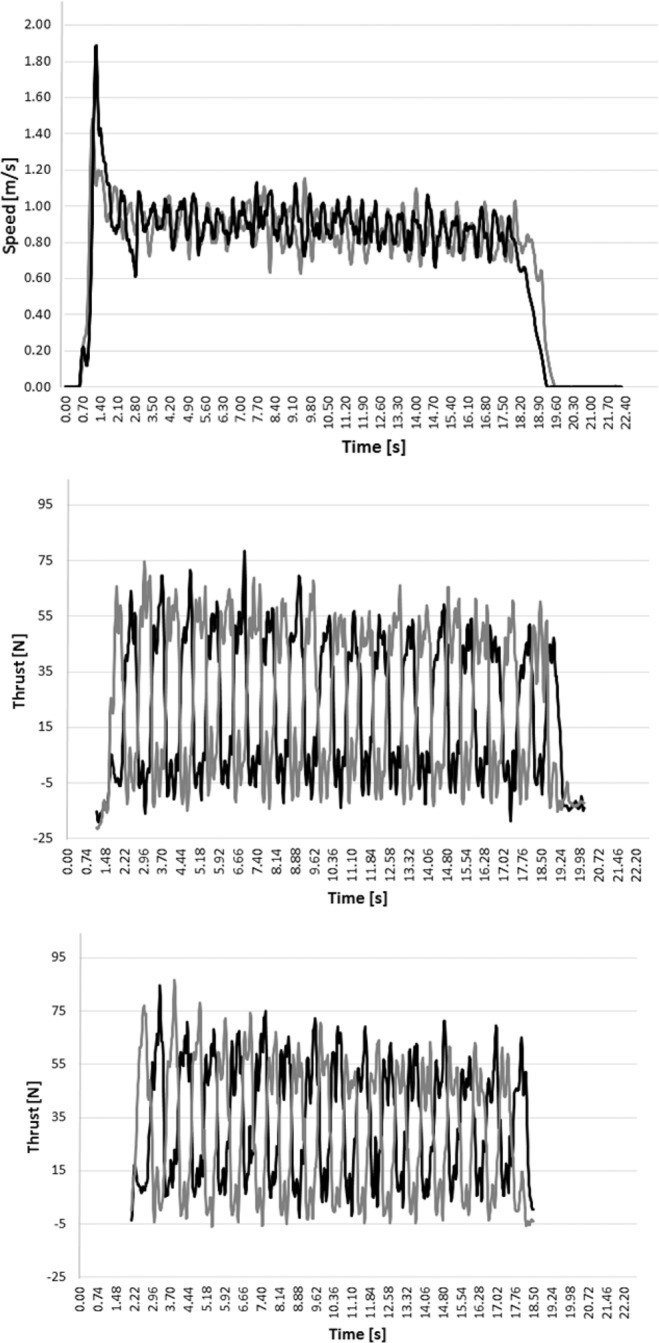
The typical time-series of one participant. Top panel: Black line depicts the body’s speed in PAP condition, whereas grey line non-PAP condition. Middle panel: Black line depicts left hand thrust and grey line right hand in non-PAP condition. Bottom panel: Black line depicts left hand thrust and grey line right hand in PAP condition.

Figure 1 depicts the typical time-series of body’s speed and arm-pull thrust by one participant in both trials (PAP and non-PAP). There was a large improvement of the arm-pull kinetics after completing the warm-up with PAP sets (0.50 < d < 0.74) (Table 1). Worthwhile change between conditions was expected to be between 3.20% (in the case of peak thrust) and 5.54% (mean thrust). However, the percentage of individual change was much larger, ranging between 13.37% (peak thrust: *P* = 0.010, d = 0.74) and 18.90% (mean thrust: *P* = 0.054, d = 0.50). The bootstrapped 95CI of the peak thrust moved from 66.1–78.7N b and to 74.5–87.9N band. Likewise, the bootstrapped 95CI of the mean thrust moved from 23.8–32.4N to 26.6–36.7N, and of the thrust-time integral from 28.7–38.2N.s to 34.4–41.8N.s. Therefore, it was noted a meaningful improvement in arm-pull thrust after undergoing PAP warm-up.

**Table 1:** The effect of post-activation potential (PAP) on arm-pull kinetics and kinematics.

	Non-PAP Mean±SD (95CI)	PAP Mean±SD (95CI)	Worthwhile change (% of non-PAP)	Δ	*P*	|d|
Peak thrust [N]	72.3 ± 11.6	80.9 ± 11.9	2.32N	13.37%	0.010	0.74
	(66.1–78.7)	(74.5–87.9)	(3.20%)			
Mean Thrust [N]	27.9 ± 7.7	31.9 ± 8.1	1.55N	18.90%	0.054	0.50
	(23.8–32.4)	(26.6–36.7)	(5.54%)			
Thrust-time integral [N.s]	33.5 ± 8.6	38.3 ± 6.2	1.25N.s	18.73%	0.004	0.63
	(28.7–38.2)	(34.4–41.8)	(3.26%)			
Speed [m/s]	0.84 ± 0.10	0.86 ± 0.09	0.02 m/s	2.78%	0.307	0.18
	(0.78–0.89)	(0.80–0.90)	(2.08%)			
Speed fluctuation [dimensionless]	0.14 ± 0.02	0.14 ± 0.05	0.01	0.73%	0.498	0.04
	(0.12–0.15)	(0.11–0.17)	(7.41%)			

SD – standard deviation, 95CI – bootstrapped 95% confidence interval, Δ – percentage of individual change, *P* – P-value, d – Cohen’s d.

There were non-significant and small effects of PAP on speed (*P* = 0.307, d = 0.18) and speed fluctuation (*P* = 0.498, d = 0.04) (Table 1). Worthwhile changes in speed and speed fluctuation were expected to be 2.08% and 7.41%, respectively. However, the percentage of mean individual change was just slightly higher in the case of speed (Δ = 2.78%, i.e. 0.70% above the expected worthwhile cut-off) and much smaller in the case of speed fluctuation (Δ = 0.73%, i.e. 6.68% away of the estimated 7.41%). Moreover, there is almost an overlap of the bootstrapped 95CI in both conditions in these two kinematic variables. As such, there is a trivial or small effect of PAP on speed and almost null on speed fluctuation.

All correlation coefficients between thrust variables were significant with a strong association, except the pairwise mean thrust vs. peak thrust in PAP that was non-significant but yet on the lower limit of a strong association (Table 2). Pooling the data (i.e. merging PAP and non-PAP datasets) the association between mean thrust and peak thrust was R = 0.69 (*P* < 0.001), between mean thrust and thrust-time integral R = 0.86 (*P* < 0.001), and between peak trust and trust-time integral R = 0.77 (*P* < 0.001). Hence, there is a significant and strong association among different kinetic variables, albeit the proportion of the variance is about 50–75% for pooled data.

**Table 2:** Correlation matrix of the association among the kinetic variables during arm-pull. PAP - post-activation potential, *P* – P-value.

		Mean Thrust	Peak Thrust	Thrust-time integral
Non-PAP	Mean Thrust	1.00		
	Peak Thrust	0.82 (*P* = 0.001)	1.00	
	Thrust-time integral	0.90 (*P* < 0.001)	0.76 (*P* = 0.004)	1.00
PAP	Mean Thrust	1.00		
	Peak Thrust	0.51 (*P* = 0.088)	1.00	
	Thrust-time integral	0.81 (*P* = 0.001)	0.74 (*P* = 0.006)	1.00
Pooled data (Non-PAP + PAP)	Mean Thrust	1.00		
	Peak Thrust	0.69 (*P* < 0.001)	1.00	
	Thrust-time integral	0.86 (P < 0.001)	0.77 (*P* < 0.001)	1.00

## Discussion

The aim of this study was to analyse the front-crawl arm-pull kinetics and kinematics by an experimental technique, comparing the PAP effect, and the associations between variables assessing the arm-pull thrust. The key findings were that after PAP sets, there is a large improvement in arm-pull thrust (about 13% to 19%) and a small improvement in performance (almost 3%). Variables commonly used to characterise thrust are strongly correlated (50–75% of variance).

Using differential pressure sensors on a triathlete, swimming at 0.8 m/s, thrust was noted as ranging between 20–40 N with each arm-pull^14^. In another study, selecting the same set-up, but at 0.90 m/s, authors reported peak force ranging between 35–50 N^17^. The peak force of an US Olympic champion, swimming at 1.66 m/s, was estimated to be 175 N by 3D video analysis and vector computation^32^. In another study, also on an US Olympic champion, but not reporting the swim speed, the peak thrust in the upsweep was 29.6 lb (i.e., 134N)^33^. Conversely, using a tethered technique, the mean thrust and peak thrust were 39N and 158N, respectively^33^. A coupled biomechanical–smoothed particle hydrodynamics fluid model estimated a peak force of 250–300 N at 1.45–1.47 m/s, on a highly-skilled Australian swimmer^34^. Therefore, if benchmarked with literature, and having as reference the competitive level of the swimmers recruited and the swim speed, our thrust values are within the expected range.

There was a large improvement of the arm-pull kinetics after completing the warm-up with PAP sets (0.004 < *P* < 0.054, 0.50 < d < 0.74). Worthwhile change was expected to range from 3.20% to 5.54%. The percentage of individual change was larger than this, being between 13.37% and 18.90%. In addition, the bootstrapped 95CI clearly shifted to the right, denoting an obvious increase in thrust production after PAP condition. A significant enhancement of kicking trust by 15% (0.40 < d < 0.66) was also reported in flutter kick after PAP conditioning sets consisting of unloaded countermovement jumps^16^. Hence, the relative improvement and standardised effect of PAP on arm-pull is similar to kicking.

Upper-limbs thrust (T_h_) can be modelled as^35^:

3 $${T}_{h}={\int }_{0}^{l}0.5\cdot \rho \cdot {({v}_{R}\cdot [-10])}^{2}\cdot Wth(r)\cdot {C}_{D}(r)dr$$

where *ρ* is water density, *v*_*R*_ the upper limb’s velocity, *Wth(r)* the upper-limbs area (i.e. the width along the length of the upper-arm), *C*_*D*_*(r)* the upper-limb’s drag coefficient. One can argue that there are no significant changes in all these terms, except the *v*_*R*_. There is no change in ρ, *Wth(r)* and *C*_*D*_*(r)*, unless if the swimmer makes significant changes in pitch angles^7^. Thus, the increase in *T*_*h*_ must be related mostly to an increase in the limb’s velocity, which in turn, can be due to an acute enhancement of the neuromuscular mechanism.

PAP phenomenon is underpinned by a few mechanisms: (i) the phosphorylation of myosin regulatory light chains, causing the actin-myosin to be more sensitive to calcium released from the sarcoplasmic reticulum in follow-up muscle contractions and, therefore an increase in force tension^36^; (ii) increased synaptic excitation within the spinal cord, resulting in enhanced post-synaptic potentials, and hence increased force tension^37^; (iii) decreased pennation angle, increasing the mechanical advantage and force transmission to tendon-bone structures^20^; (iv) increased compliance by connective tissue and tendons^20^. Altogether, a meaningful improvement in arm-pull thrust was noted after the PAP conditioning sets.

There were non-significant and small effects of PAP on speed and almost null effects on speed fluctuation (0.307 < *P* < 0.498, 0.04 < d < 0.18). The percentage of individual change in speed was 2.78%, whereas the estimated worthwhile change was 2.08%. Hence, there was a small effect of PAP on swim speed. If a sprinter races the 100 m freestyle in 50 s, a 2.5–3.0% improvement in performance translates to a 0.98–1.25 s reduction in the final race time. Converting a d=0.18 to percentile gain, it represents a 7% improvement. I.e., everything else being equal, undergoing PAP can lead to moving up 7 places in a ranking featuring 100 contenders. For instance, if one is within the top-16 out of 100 contenders in a swimming event, undergoing PAP makes the sprinter jump to top-9, increasing the likelihood of going through the finals (top-8). The bootstrapped 95CI band from non-PAP to PAP shifted from 0.78–0.89 m/s to 0.80–0.90 m/s. Hence, there is 95% confidence that speed will increase by 0.01–0.02 m/s. Swimming the 100 m in 50 s, the average speed is 2.0 m/s. Increasing the average speed to 2.01 m/s and 2.02 m/s, the final race time will be 49.75 s and 49.50 s, respectively. Altogether, even though the small effect of PAP on speed, this should not be overlooked because it can have a meaningful impact on swimmer’s performance.

The thrust-time series can be decomposed into several variables to analyse and report the kinetics. Same procedure is carried out in time-series of on-land kinetic variables. Such as, ground reaction force^28^ and plantar pressure^29–31^. Due to the large amount of variables that it is possible to extract, one may wonder how redundant are they. I.e., if these variables can be interpreted interchangeably. All correlation coefficients were significant with a strong association, up to 81% of variance. For pooled data (PAP plus non-PAP sub-datasets) the variance ranged between 50 and 75%. The range of Pearson’s Correlation Coefficients match the values reported for plantar pressures (0.78 < R < 0.90)^29^. Another study also reported strong associations (R > 0.78) between peak pressure and pressure-time integrals^30^. A systematic review noted that the value of reporting pressure–time integral in addition to peak pressure data is limited in dynamic plantar pressure studies on diabetic foot^31^. Hence, for both human locomotion on-land and in-water, the report of the impulse (force-time or pressure-time integrals) is redundant to straightforward parameters such as peak and mean values. In some settings, such as when researchers or practitioners are facing time constrains or running the test on a large number of subjects, the report of a smaller set of variables (peak and mean values) can be very convenient. Also, the interpretation of findings across studies that report different variables is a possibility.

Two of the main findings were that after PAP conditioning sets thrust had a large improvement, whereas speed a small improvement. I.e., the proportion of improvement by performance (~2.5–3.0%) does not match the proportion of thrust enhancement (~13–19%). Thrust improved five times more than performance. Thus, it begs the question why there is a disproportional improvement in performance and thrust. In a study on the effect of PAP on flutter kick, thrust improved by 15% and performance ~10%^16^. It seems the efficiency of the transfer of thrust into speed is larger for kicking than for performing arm-pulls. Hence, one may wonder why is the efficiency smaller during arm-pulls. Equation 2 denotes the main swimming determinants. These encompass the thrust by lower-limbs and upper-limbs, the three drag components (friction drag, pressure drag and wave drag) and the mass to be carried out (body mass and added mass of water). As noted early on, both upper- and lower-limbs thrust increased. The added mass of water depends on the body anthropometrics, that remains unchanged^38^. As such, the disproportional improvement of speed with larger thrust might be explained by an increase of the drag force. Indeed swimming faster leads to larger drag force (D = k.v^2^). Friction drag (D_f_) can be estimated as^3^:

4 $${D}_{f}=0.5\cdot \rho \cdot {v}^{2}\cdot {A}_{wetted}\cdot {C}_{Df}$$

and,

5 $${A}_{wetted}=0.20247\cdot {L}^{0.725}\cdot B{M}^{0.425}$$

6 $${C}_{Df}=\frac{0.075}{{(\log (Re)-2)}^{2}}$$

7 $$Re=\frac{v\cdot L}{\vartheta }$$

where *ρ* is the density of the water, *v* the velocity, *A*_*wetted*_ the wetted surface area, and *C*_*Df*_ the friction drag coefficient, *L* the body length, *BM* the body mass, *Re* the Reynolds number, and υ the water kinematic viscosity. So, the increase in velocity leads to an increase in *D*_*f*_, as other terms will remain unchanged. Pressure drag (*D*_*p*r_) is assumed as being related to bluff body separation, with viscous pressure resistance due to negligible boundary layer growth^3^:

8 $${D}_{pr}=0.5\cdot \rho \cdot {v}^{2}\cdot S\cdot {C}_{Dpr}$$

where *ρ* is the density of the water, v the velocity, *S* the trunk transverse surface area, and *C*_*Dpr*_ the pressure drag coefficient. Again, most changes in *D*_*pr*_ are due to velocity and maybe *C*_*Dpr*_, for instance, if there is any change in the limb’s 3D motor path underwater or body position that we did not monitored. Lastly, the wave drag (*D*_*w*_):

9 $${D}_{w}=0.5\cdot \rho \cdot {v}^{2}\cdot {A}_{wetted}\cdot {C}_{Dw}$$

where *ρ* is the density of the water, *v* the velocity, *A*_*wetted*_ wetted surface area, and *C*_*Dw*_ the wave drag coefficient. *C*_*Dw*_ is strongly dependent on Froude number:

10 $$Fr=\frac{v}{\sqrt{g\cdot L}}$$

where *v* is the velocity, *g* the local gravitational acceleration and *L* the body length. Hence, the speed increase leads to larger *Fr*, *C*_*Dw*_ and *D*_*w*_. Moreover, *D*_*w*_ accounts to 50% of total drag force^39^. Thus *D*_*w*_ might have played a key-role increasing the resistance to displacement and, therefore, ultimately in the smaller performance improvement.

The following limitations can be pointed out for this research: (1) the effect of different latency periods in the findings is not known; (2) the effect of PAP in other swim strokes (e.g., backstroke, breaststroke, butterfly stroke) is still unknown; (3) whether the PAP effect will be the same for distances longer than 25 m requires further investigation; (4) the system is only able to measure the normal component of the thrust vector, instead of the effective propulsive force (i.e. the propulsive force in the direction of body´s displacement). To report the effective propulsive force (i.e. the component of the thrust vector in the direction of displacement) one would need to run either a 3D kinematic analysis or, alternatively, to place IMUs on the hand/wrist. Having said that, this research is a crossover design and as such, the assumption is that in both conditions there is a not significant change in the hands’ underwater motor path and orientation. There are no reasons to believe that PAP would induce changes in the 3D motor path and hands’ orientation as compared to non-PAP.

In conclusion, after undergoing a warm-up that includes PAP sets for upper-body, there is a large improvement in arm-pull thrust and a small improvement in performance. Variables most commonly used to characterise thrust are strongly correlated and as such can be used interchangeably reporting the arm-pull thrust. PAP conditioning sets in the form of resistance band arm-pulls elicit large increases of thrust production that in turns, leads to a small enhancement of the performance.

## Methods

### Design

A randomised crossover research design was selected to compare the effects of a standard warm-up without a PAP set (non-PAP; control condition) and another with PAP sets (PAP; experimental condition) in the arm-pull kinetics, kinematics and performance. Participants attended three sessions. One familiarisation session with testing procedures and selection of the resistance band level. Then, one testing session of each condition. The first testing session took place 48 hours after familiarisation and second testing session one week after the first testing session. In each testing session, participants performed randomly one of the two conditions (non-PAP or PAP warm-ups) followed-up by a 25 m all-out trial in front-crawl arm-pull, with push-off start. Latency period between end of warm-up routine and in-water time trial was set at 8 minutes^40^.

### Participants

Twelve skilful local male competitive swimmers were recruited (23.50 ± 3.35 years of age, 70.97 ± 7.91 kg of body mass, 1.76 ± 0.04 m tall, 8.08 ± 4.59 years of competitive experience). The inclusion criteria comprised: (1) males; (2) competitive swimmers; (3) racing at local, national or international competitions in the past. Exclusion criteria was as follows: (1) non-competitive swimmer (e.g. water polo players); (2) suffering from any injury or disease in the past six months; (3) unable to attend the three scheduled sessions of this study.

All procedures performed in studies involving human participants were in accordance with the ethical standards of the institutional and/or national research committee and with the 1964 Helsinki declaration and its later amendments or comparable ethical standards. The Institutional Review Board of the Nanyang Technological University approved the study. All participants had been briefed about their rights before signing a written informed consent form. In the case of under-age participants, parents and/or legal guardians were also briefed before signing a written informed consent.

### Warm-up routines

Participants were randomly assigned to perform two different warm-ups in a crossover manner: (1) non-PAP (control condition) and (2) PAP (experimental condition). Latency period between each warm-up and carrying out in-water testing was set at 8 minutes^16,22,41^. For instance, it was compared the effects of PAP on swim start (time to 15 m) after ~15 s, 4, 8, 12 and 16 minutes in a group of international sprint swimmer. It was noted that best performance was delivered after a latency period of 8 minutes^41^. Warm-ups were designed based on past evidence and coaches’ experience^16,42–44^.

The total mileage for non-PAP warm-up was set at 1,400 m. The warm-up consisted of swimming 400 m in self-selected stroke and pace (i.e., any stroke and speed of choice), 200 m of front-crawl drills (25 m steady/25 m fast), 200 m of flutter kick drills using a kickboard (15 m fast/35 m steady), 4 × 100 m (2 front-crawls and 2 individual medleys with 10 s rest in between), 100 m (easy) and 2 × 50 m (dive followed by 15 m fast/35 m easy) of front-crawl drills.

In the PAP warm-up, participants were required to perform 700 m plus PAP sets to match the overall workload of non-PAP condition. I.e. warm-up routine was the same as in non-PAP condition, but performing half of its mileage. The warm-up consisted of swimming 200 m in self-selected stroke and pace, 100 m of front-crawl drills (25 m steady/25 m fast), 100 m of flutter kick drills using a kickboard (15 m fast/35 m steady), 2 × 100 m (1 front-crawl and 1 individual medley with 10 s rest in-between), 50 m (easy) and 50 m (dive followed by 15 m fast/35 m easy) of front-crawl drills. To induce PAP, in-water warm-up was followed-up by 5 minutes of rest before performing on-land 2 sets of 5 maximal repetitions of resistance band pull with 2 min of rest between sets by each upper-arm^40,45^. Resistance band level was chosen on individual basis during the familiarisation session (light-medium, medium or heavy; resistance range: 3.17–19.50 kg, 4.53–22.68 kg and 7.27–26.76 kg, respectively).

### In-water testing

Participants performed the in-water testing 8 min after completing each warm-up. The in-water testing was a 25 m all-out bout, using arm-pull in front-crawl, while lower-limbs were held by a pull-buoy. Participants were instructed to minimise gliding after push-off from headwall.

Kinetics and kinematics were collected by an in-house customised system integrating and synchronising data from differential pressure sensors (Aquanex, Swimming Technologies, Florida, USA), speedo-meter (Swim speedo-meter, Swimsportec, Hildesheim, Germany) and underwater camera (Aquanex, Swimming Technology Research, Inc., USA). Differential pressure sensors were placed between 3^rd^ and 4^th^ proximal phalanges and metacarpals of each hand. It is assumed this place as being a good proxy of the application point of the thrust vector on the hand^46,47^. A larger number of sensors on each hand can lead to technique constraints because of the cabling surrounding the upper-limb. Also, increasing the number of sensors it might change the geometry and volume of the hand, having an impact on the ecological validity of the data. The sensors measure the change in pressure between inlet and outlet and then force is derived. In our case, it is measured the pressure of the water on the dorsal (low pressure field) and palmar (high pressure field) surfaces, being computed the difference between both. There is a diaphragm inside the sensor that flexes and is sensed as an electrical signal that is proportional to the difference in the two pressures. Accuracy of the system was reported elsewhere^48^. The speedo-meter was set on the headwall of the pool, about 0.2 m above water surface. The string of the speedo-meter was attached to a belt worn on the waist. Underwater camera was set-up 0.5 m deep on the headwall, providing an underwater view in the transverse plane.

A customised software (LabVIEW®, v.2017) was used to collect (*f* = 50 *Hz*), streaming and playback time-series data, as well as, video signal of each trial. Data was transferred from different components of the system (i.e., pressure sensors, speedo-meter and underwater camera) to software interface by a 14-bit resolution acquisition card (NI-6001, National Instruments, Austin, Texas, USA). Then, data was imported into a signal processing software (AcqKnowledge v. 3.9.1, Biopac Systems, Santa Barbara, USA). Fourteen arm-pulls by each upper-limb (overall: 28 arm-pulls) were analysed and mean values for selected kinetic and kinematic parameters were calculated for further analysis.

**Figure 2:**
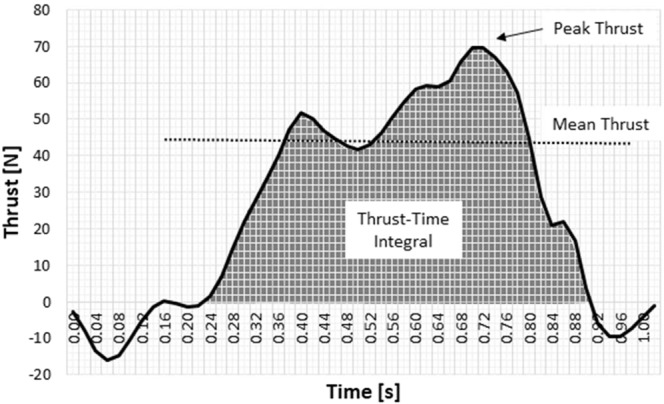
The typical thrust-time plot of an arm-pull in front-crawl and the selected kinetic variables.

Multiplying the pressure by the area, the thrust is then calculated^49^. The kinetic variables analysed included the peak thrust (i.e., the maximal value, in N), mean thrust (in N) and thrust-time integral (in N.s) (Fig. 2). Speed (in m/s) and speed fluctuation (dimensionless) were selected as kinematic variables. Speed fluctuation can also be deemed as a proxy of energy cost. It was reported by analytical procedures and experimental testing that there is an inverse relationship, in full stroke swimming, between speed fluctuation and energy cost of transportation^50^.

### Statistical analysis

Mean ± standard deviation (SD) and mean percentage of individual change are reported for all dependent variables. Uncertainty in each condition (i.e. independent variables) was computed by bootstrapping 95% confidence intervals (95CI) (1,000 samples).

Randomisation test was run to compare the differences between conditions (paired samples, 20 permutations, 1,000 repetitions, *P* ≤ 0.05). Cohen’s d was selected as standardised effect size of mean differences and deemed as: (i) |d | < 0.2 trivial; (ii) 0.2 < |d | ≤ 0.5 medium; (iii) |d | > 0.5 large.

Between-subjects worthwhile changes in control condition (non-PAP) were computed to examine the smallest meaningful improvement required when undergoing PAP. Worthwhile change was calculated by having *d* = 0.2 as the smallest standardized effect size in sports performance^51^. Then, worthwhile change was converted into smallest partial improvement to be expected having as reference the mean value of non-PAP condition (i.e., the smallest meaningful percentage of change expected to be meaningful from control to experimental conditions).

Pearson’s Correlation Coefficients among the three kinetic variables (peak thrust, mean thrust and thrust-time integral) were computed to assess the levels of association (*P* ≤ 0.05). Correlation effect sizes were deemed as: 0 < |R | ≤ 0.1 null; 0.1 < |R | ≤ 0.3 small; 0.3 < |R | ≤ 0.5 moderate; |R | > 0.5 strong. Statistical analyses were run on R.

## Data Availability

The datasets generated and/or analysed during the current study are available from the corresponding author on reasonable request.
